# Short-term efficacy of angiotensin receptor-neprilysin inhibitor treatment in patients with ST-segment elevation myocardial infarction with reduced ejection fraction after primary percutaneous coronary intervention: a propensity score matching study

**DOI:** 10.1186/s12872-022-02906-0

**Published:** 2022-11-04

**Authors:** Qun Zhang, Bao Qiao, Yu Han, Shukun Sun, Bailu Wang, Shujian Wei

**Affiliations:** 1 Department of Emergency and Chest Pain Center, Qilu Hospital of Shandong University, Jinan, 250012 Shandong China; 2 Clinical Research Center for Emergency and Critical Care Medicine of Shandong Province, Qilu Hospital of Shandong University, Jinan, 250012 Shandong China; 3 Key Laboratory of Emergency and Critical Care Medicine of Shandong Province, Key Laboratory of Cardiopulmonary-Cerebral Resuscitation Research of Shandong Province, Qilu Hospital of Shandong University, Jinan, 250012 Shandong China; 4 Clinical Trial Center, Qilu Hospital of Shandong University, Jinan, 250012 Shandong China

**Keywords:** Ventricular remodeling, ST-segment elevation myocardial infarction, Angiotensin receptor-neprilysin inhibitor, Angiotensin-converting enzyme inhibitor, Primary percutaneous coronary intervention

## Abstract

**Background:**

Acute myocardial infarction (AMI) causes a series of pathophysiological changes, including myocardial necrosis, myocardial edema, and microvascular damage. These changes eventually lead to severe cardiovascular events, such as ventricular remodeling, heart failure, and papillary dysfunction. Impaired cardiac function after ST-segment elevation myocardial infarction (STEMI) often manifests as a decrease in left ventricular ejection fraction (LVEF). Clinical trials have shown that angiotensin receptor-neprilysin inhibitor (ARNI) treatment has the potential to improve LVEF in patients with STEMI after primary percutaneous coronary intervention (PPCI).

**Objective:**

The purpose of this study was to evaluate the short-term efficacy of ARNI versus angiotensin-converting enzyme inhibitor (ACEI) treatment in patients with STEMI who exhibit reduced LVEF after PPCI.

**Methods:**

A total of 169 patients with STEMI exhibiting post-PPCI LVEF below 50% who were orally treated with ARNI between December 2017 and August 2020 were selected as the experimental group. A total of 136 patients with STEMI exhibiting post-PPCI LVEF below 50% who were orally treated with an ACEI between January 2016 and August 2020 were selected as the control group. LVEF was measured using cardiac ultrasonography during hospitalization and 3 months after discharge. Linear and logistic regression analyses were performed to compare patient demographics and hospitalization variables to evaluate the risk factors for change and rate of improvement in LVEF. Propensity score matching (PSM) was used to account for confounding factors.

**Results:**

After PSM, the study cohort consisted of 81 patients in the ARNI group and 123 in the ACEI group. After an average follow-up period of 3 months, no significant difference was noted in the LVEF improvement rate between the experimental and control groups (*P* = 0.475, 95% CI: -0.062 to 0.134). Multivariate logistic regression analysis also indicated no significant correlation between the change in LVEF and oral ARNI treatment in patients with STEMI exhibiting reduced LVEF after PPCI (*P* > 0.05).

**Conclusion:**

The short-term effect of ARNI treatment on the cardiac function of patients with STEMI and reduced LVEF after PPCI is not superior to that of ACEI treatment.

**Supplementary Information:**

The online version contains supplementary material available at 10.1186/s12872-022-02906-0.

## Introduction

Sacubitril-valsartan is an angiotensin receptor-neprilysin inhibitor (ARNI) with the dual actions of inhibiting enkephalinase and antagonizing angiotensin receptors [1]. On the one hand, valsartan can inhibit the renin-angiotensin–aldosterone system, reduce cardiac load, relax blood vessels, and delay or reverse ventricular remodeling. On the other hand, the ARNI can improve myocardial remodeling, diuresis, and sodium excretion by enhancing the natriuretic peptide system through the action of sacubitril [1, 2]. Since the development of primary percutaneous coronary intervention (PPCI), the treatment success rate for acute myocardial infarction (AMI) has been greatly improved; however, the prevention of heart failure (HF) after AMI is still worthy of clinicians’ attention.

AMI can cause severe damage to heart function, affecting the daily activities of patients and creating a burden on society [3]. Patients with HF after AMI still have a high mortality rate even with angiotensin-converting enzyme inhibitor (ACEI) treatment. ARNI can improve myocardial remodeling by preventing cardiomyocyte death, hypertrophy, and cardiomyocyte contraction [4, 5]. Previous studies have demonstrated the potential advantages of ARNI in the treatment of AMI [6, 7]. ARNI has a direct protective effect on the myocardium after MI, andis more effective than ACEIs in inhibiting left ventricular remodeling [8]. Animal experiments show that, compared with ACEI drugs, ARNI can better reverse myocardial remodeling after AMI and improve other indicators such as the left atmospheric volume index [9] and left ventricular ejection fraction (LVEF) [4].

At present, few relevant clinical trials have focused on the therapeutic efficacy of ARNI and ACEIs for patients with STEMI after PPCI. Therefore, it is necessary to evaluate the efficacy of ARNI and ACEIs in the treatment of patients with STEMI. In this study, we retrospectively analysed the short-term effect of oral ARNI vs. ACEI treatment on STEMI patients with reduced LVEF after PPCI.

## Materials and methods

### Subjects

In this retrospective analysis, we utilized data from 305 patients with STEMI who exhibited reduced LVEF after PPCI. A total of 136 patients treated with oral ARNI during hospitalization at Qilu Hospital of Shandong University (Jinan, Shandong Province, China) between December 2017 and August 2020 were selected as the experimental group. A total of 169 patients who had been orally treated with ACEIs, such as perindopril and benazepril, at the same hospital between January 2016 and August 2020 were selected as the control group. Patients were followed up for an average of 3 months.

### Inclusion and exclusion criteria

The inclusion criteria for patients were as follows: (1) meeting STEMI diagnostic criteria; (2) classification as type 1 MI; (3) LVEF < 50%; and (4) undergoing PPCI treatment.

The exclusion criteria for patients were as follows: (1) a history of drug allergies and contraindications; (2) severe impairment of liver and kidney function; (3) systolic blood pressure < 90 mmHg; (4) diagnosis of a mental disorder and inability to cooperate; and (5) hyperkalaemia and malignant tumors.

### Definition

The diagnostic criteria of STEMI are as follows: (1) typical manifestations of ischemic chest pain; (2) electrocardiogram-detected hyperacute T-waves with (typically arcuate dorsal) ST-segment elevation and mirrored ST-segment depression of corresponding leads; (3) troponin positivity; and (4) imaging evidence of coronary angiography [10].

### Selection of baseline characteristics

The following variables were selected for analysis: demographic data (including age and sex); comorbidities (including diabetes and hypertension); smoking and drinking history; LVEF and medication during hospitalization; and self-reported history of diabetes, fasting blood glucose ≥ 7.0 mmol/l, and insulin and/or oral hypoglycemic drugs [11]. Last, we recorded patients’ self-reported medical histories of antihypertensive drug use, the average of two resting systolic blood pressure readings ≥ 140 mm Hg, and/or the average of two diastolic blood pressure readings ≥ 90 mm Hg [12].

### Follow-up plan

Follow-up patient information was obtained by outpatient contact via telephone and other means. The average follow-up time was 3 months. The follow-up data collected mainly comprised LVEF measurements.

### Clinical outcome

The main variables measured in each group were the change and improvement rate in LVEF. The change in LVEF was calculated as follows:

$${LVEF}_{change}={LVEF}_{follow-up}-{LVEF}_{Hospitalization}$$

The LVEF improvement rate was subsequently calculated as follows:

$${LVEF}_{improvementrate}=\frac{{LVEF}_{change}}{{LVEF}_{Hospitalization}}$$

### Propensity score matching

We performed propensity score matching (PSM) between the experimental and control groups to control for bias and minimize the effect of confounding factors. Matching covariates consisted of sex, age, diabetes, hypertension, smoking and drinking history, and medication during hospitalization. A 1:2 matching protocol with a greedy matching algorithm was used, and the caliper width of the logit standard deviation of the propensity score was 0.05.

### Statistical analyses

A multiple-interpolation method was used first to process missing data for continuous and binary variables. Categorical data are presented as percentiles, whereas continuous variables with normal and nonnormal distributions are presented as the mean ± standard deviation and median (interquartile range), respectively. The chi-squared test and Fisher’s exact test were used to compare categorical data between the groups, and the nonparametric Mann–Whitney U test and independent sample Student’s *t test* were used to compare continuously distributed data between the groups. Linear and logistic regression models were constructed to screen the risk factors for LVEF improvement rate and change, respectively. Multivariate logistic regression models were used to estimate the odds ratio and 95% confidence interval to determine any association between LVEF (change) and risk factors.

## Results

### Baseline characteristics

**Fig. 1:**
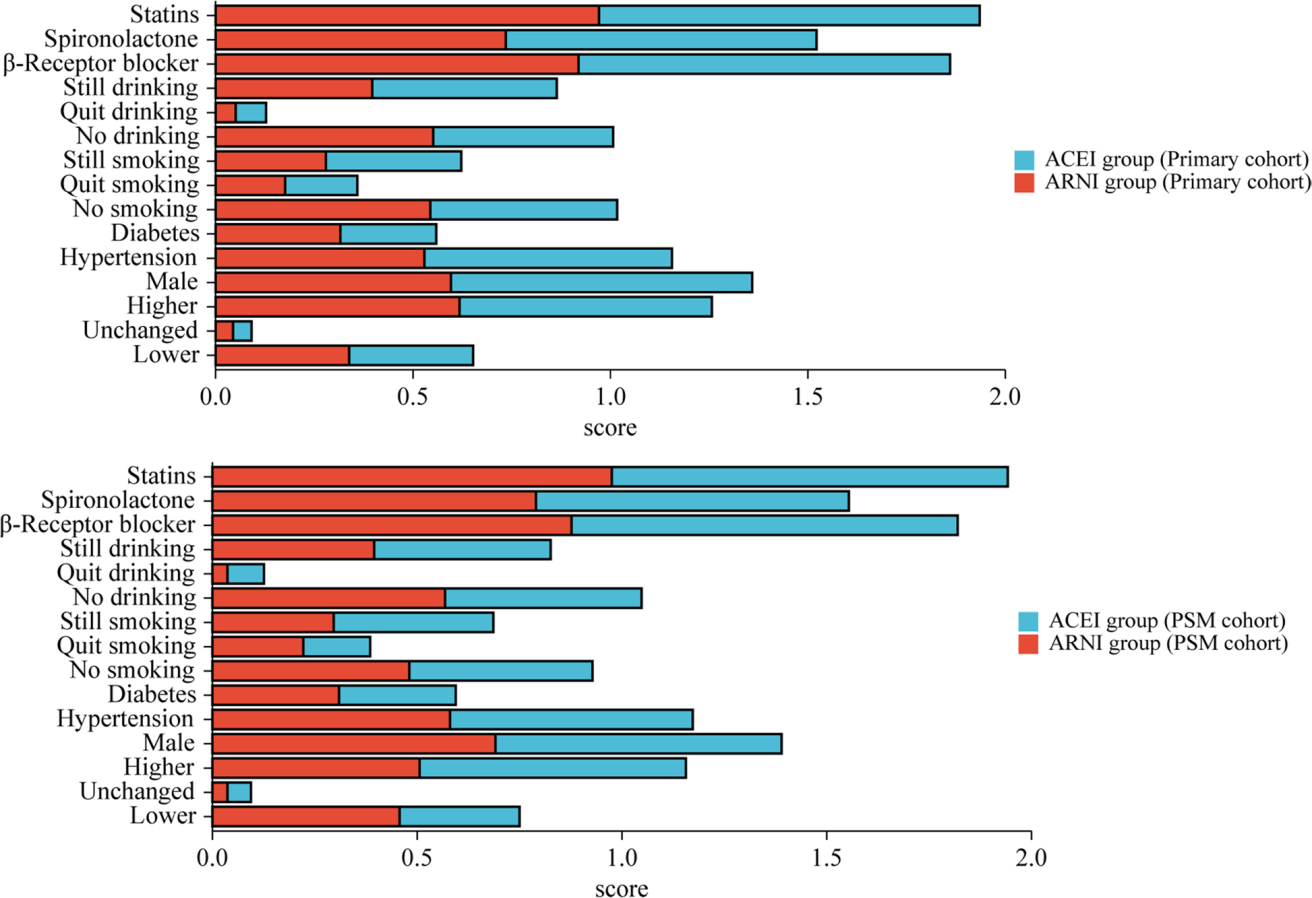
The bar chart of categorical data

**Fig. 2:**
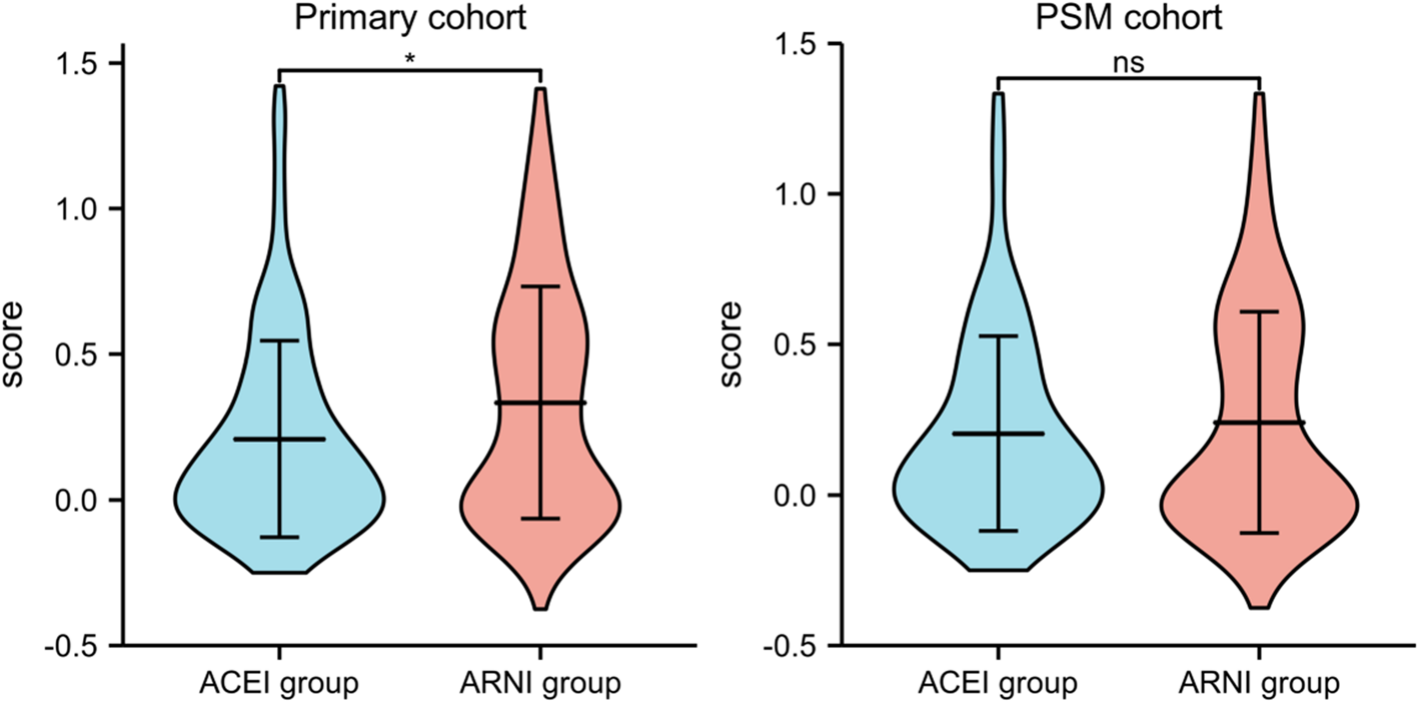
The Violin plot of the LVEF (improvement rate)

The clinical characteristics of the 305 patients with STEMI monitored in this study are listed in Table 1. Patients were nonhomogenous across multiple variables. However, no significant difference in variables other than age and gender was noted between the control and experimental groups. After PSM, the cohort consisted of 81 patients in the ARNI group and 123 in the ACEI group (Supplemental Fig. 1). No significant difference was found across all variables. The bar chart of categorical data is shown in Fig. 1. The violin plot of the LVEF (improvement rate) is shown in Fig. 2.

**Table 1:** The demographics and clinical characteristics of the subjects

	**Primary cohort**				**PSM cohort**			
	**Experimental Group**	**Control Group**	B	***P* value**	**Experimental Group**	**Control Group**	B	***P* value**
N	136	169			81	123		
Age (years), M (P25, P75)	60(53,66)	69(64,72)	-8.983	< 0.001	66(61,68)	66(63,70)	-2.225	0.026
Sex			9.885	0.002			0.014	0.905
Male, n(%)	81(59.6)	129(76.3)			56(69.1)	86(69.9)		
Female, n(%)	55(40.4)	40(23.7)			25(30.9)	37(30.1)		
Hypertension, n(%)			2.966	0.085			0.035	0.851
Yes	72(52.9)	106(62.7)			47(58)	73(59.3)		
No	64(47.1)	63(37.3)			34(42)	50(40.7)		
Diabetes, n(%)			2.044	0.153			0.137	0.712
Yes	43(31.6)	41(24.3)			25(30.9)	3528.5)		
No	93(68.4)	128(75.7)			56(69.1)	88(71.5)		
Smoking history, n(%)			1.741	0.419			2.278	0.320
No smoking	74(54.4)	80(47.3)			39(48.1)	55(44.7)		
Quit smoking	24(17.6)	31(18.3)			18(22.2)	20(16.3)		
Still smoking	38(27.9)	58(34.3)			24(29.6)	48(39)		
Drinking history, n(%)			2.990	0.224			2.843	0.241
No drinking	75(55.1)	77(45.6)			46(56.8)	59(48)		
Quit drinking	7(5.1)	13(7.7)			3(3.7)	11(8.9)		
Still drinking	54(39.7)	79(46.7)			32(39.5)	53(43.1)		
NT-proBNP, M (P25, P75)	1370(656.13,2681)	1519(703.9,2920)	-0.696	0.486	1571 (803.2, 2752)	1551 (775,2749)	-0.175	0.861
CTNI, M (P25, P75)	229.98(42.56,1877.78)	469.99(20.76,4719.5)	-1.033	0.302	223.4 (40.72)	714.52 (19.3,4894)	-1.582	0.114
β Receptor blocker, n(%)			0.554	0.457			2.831	0.092
Yes	125(91.9)	159(94.1)			71(87.7)	116(94.3)		
No	11(8.1)	10(5.9)			10(12.3)	7(5.7)		
Spironolactone, n(%)			1.116	0.291			0.188	0.665
Yes	100(73.5)	133(78.7)			64(79)	94(76.4)		
No	36(26.5)	36(21.3)			17(21)	29(23.6)		
Statins, n(%)			0.088	0.767			0.105	0.746
Yes	132(97.1)	163(96.4)			79(97.5)	119(96.7)		
No	4(2.9)	6(3.6)			2(2.5)	4(3.3)		
LVEF(during hospitalization), M (P25, P75)	0.33(0.28,0.38)	0.34(0.29,0.4)	-1.180	0.238	0.35 (0.3, 0.39)	0.33 (0.29,0.4)	-0.217	0.828
LVEF(follow-up), M (P25, P75)	0.41(0.37,0.46)	0.4(0.36,0.44)	-3.102	0.002	0.4 (0.36,0.46)	0.4 (0.36,0.44)	-0.817	0.414
LVEF(change), n(%)			0.213	0.899			5.782	0.056
Lower	46(33.8)	53(31.4)			37(45.7)	36(29.3)		
Unchanged	6(4.4)	8(4.7)			3(3.7)	7(5.7)		
Higher	84(61.8)	108(63.9)			41(50.6)	80(65)		
LVEF(Improvement rate), M (P25, P75)	0.26 (-0.03, 0.62)	0.11 (-0.03, 0.39)	-2.513	0.012	0.027 (-0.05,0.55)	0.114 (-0.03,0.39)	-0.124	0.902

### Linear regression analysis

For linear regression analysis, LVEF (improvement rate) was selected as the dependent variable. Demographic data (including age and sex), comorbidities (including diabetes and hypertension), smoking and drinking history, and medication during hospitalization were selected as independent variables. After an average follow-up period of 3 months, there was no significant difference in LVEF (improvement rate) between the experimental and control groups (*P* = 0.348, 95% CI: -0.053 to 0.149). The PSM results also showed that, after the three-month follow-up period, there was no significant difference in LVEF (improvement rate) between the experimental and control groups (*P* = 0.475, 95% CI: -0.062 to 0.134). In addition, no significant correlation was found between any other variable and LVEF (improvement rate). The tolerance was greater than 0.1, and the variance inflation factor was less than 10, indicating an absence of multicollinearity (Tables 2 and 3).

**Table 2:** The Linear regression analysis of Primary cohort

Variable	B	SE	Beta	t	*P*	95%*CI*	VIF
ARNI	0.048	0.051	0.069	0.94	0.348	-0.053–0.149	1.088
Age	-0.005	0.004	-0.096	-1.254	0.212	-0.013–0.003	1.186
Sex							
Male	Ref						
Female	0.1	0.059	0.136	1.704	0.09	-0.016–0.217	1.281
Hypertension							
Yes	0.008	0.05	0.012	0.162	0.871	-0.091–0.107	1.059
No	Ref						
Diabetes							
Yes	-0.086	0.053	-0.115	-1.605	0.11	-0.191–0.02	1.035
No	Ref						
Smoking history							
No smoking	Ref						
Quit smoking	0.063	0.07	0.072	0.893	0.373	-0.076–0.201	1.299
Still smoking	0.072	0.059	0.101	1.228	0.221	-0.044–0.187	1.365
Drinking history							
No drinking	Ref						
Quit drinking	0.125	0.1	0.093	1.252	0.212	-0.072–0.322	1.109
Still drinking	0.027	0.052	0.039	0.522	0.602	-0.075–0.129	1.133
NT-proBNP	< -0.001	0	-0.046	-0.624	0.534	0	1.11
CTNI	< 0.001	0	0.061	0.82	0.413	0	1.097
β Receptor blocker							
Yes	0.062	0.09	0.051	0.694	0.489	-0.115–0.24	1.079
No	Ref						
Spironolactone							
Yes	-0.079	0.059	-0.097	-1.341	0.182	-0.195–0.037	1.052
No	Ref						
Statins							
Yes	0.043	0.146	0.021	0.295	0.768	-0.245–0.331	1.063
No	Ref

**Table 3:** The Linear regression analysis of PSM cohort

Variable	B	SE	Beta	t	*P*	95%*CI*	VIF
ARNI	0.036	0.05	0.048	0.715	0.475	-0.062–0.134	1.432
Age	-0.01	0.003	-0.223	-3.308	0.001	-0.015–0.004	1.443
Sex							
Male	Ref						
Female	0.068	0.049	0.085	1.377	0.169	-0.029–0.165	1.211
Hypertension							
Yes	0.013	0.043	0.017	0.298	0.766	-0.072–0.097	1.049
No	Ref						
Diabetes							
Yes	0.013	0.043	0.017	0.298	0.766	-0.158–0.028	1.049
No	Ref						
Smoking history							
No smoking	Ref						
Quit smoking	0.015	0.059	0.016	0.252	0.801	-0.102–0.132	1.222
Still smoking	0.02	0.052	0.026	0.393	0.695	-0.081–0.122	1.343
Drinking history							
No drinking	Ref						
Quit drinking	0.053	0.088	0.035	0.602	0.548	-0.12–0.225	1.098
Still drinking	0.034	0.044	0.046	0.783	0.434	-0.052–0.121	1.112
NT-proBNP	< -0.001	0	-0.055	-0.942	0.347	0	1.084
CTNI	< -0.001	0	-0.039	-0.686	0.493	0	1.048
β Receptor blocker							
Yes	-0.008	0.083	-0.006	-0.1	0.92	-0.173–0.156	1.043
No	Ref						
Spironolactone							
Yes	-0.055	0.05	-0.063	-1.094	0.275	-0.154–0.044	1.063
No	Ref						
Statins							
Yes	0.034	0.119	0.016	0.284	0.776	-0.201–0.269	1.055
No	Ref						

### Multivariate logistic regression analysis

For multivariate logistic regression analysis, LVEF (change) was selected as the dependent variable. Demographic data (including age and sex), comorbidities (including diabetes and hypertension), smoking and drinking history, and medication during hospitalization were selected as independent variables. Both multivariate logistic regression analysis and PSM results indicated no significant correlation between LVEF (change) and oral ARNI treatment in patients with STEMI and reduced LVEF after PPCI (*P* > 0.05). The *P* value of the likelihood ratio test of the multivariate logistic regression model was 0.040. The goodness-of-fit was 1.00, indicating that the model had a good degree of fit (Supplemental Table 1 and Supplemental Table 2).

## Discussion

The lack of a significant difference in the short-term effects between ARNI and ACEIs on LVEF may be related to the small sample size of this study. No adverse effects or deaths were recorded among patients treated with ARNI during the 3-month follow-up.

STEMI is one of the leading causes of death worldwide, and PPCI is recommended for STEMI [13]. Randomized controlled trials have shown that PPCI reduces recurrent MI and mortality more than thrombolytic therapy and significantly reduces the incidence of major in-hospital complications in patients with STEMI [14]. However, the latest data suggest that the incidence of in-hospital complications remains approximately 10% [15]. Timing plays a crucial role in ischemia–reperfusion therapy for patients with STEMI. Ideally, the time between first medical contact and PCI should not exceed 60 min, with 90 min as the upper limit [16]. However, because of various time-consuming logistical issues, varying degrees of expertise between doctors, and differences in medical equipment between hospitals, the benefits of PPCI are resticted and intravenous thrombolysis is still a preferred treatment for patients with acute STEMI in some regions. [14, 17]. Furthermore, other effort has also been devoted to improve the prognosis of AMI patients such as the CardioMEMS™ system, left ventricular assist devices, etc. Expectedly, more AMI patients should survive with the help of these new monitoring and supporting devices [18, 19].

HF is a severe complication associated with STEMI [20]. Left ventricular remodeling plays an important role in the occurrence of HF, and reversing this process has been shown to improve the patient’s prognosis [21]. Successful and timely PPCI limits the infarct size and reduces the incidence of HF. However, even with successful and timely PPCI, the incidence of left ventricular dysfunction in patients with STEMI remains high [22]. Clinical trials have shown that the important predictors of left ventricular remodeling are myocardial perfusion grade, symptom-to-portal time, and symptom-to-balloon time [23]. To some extent, PPCI is linked to the occurrence of ischemia–reperfusion injury [24]. In addition, it can lead to unpredictable no-reflow phenomenon and microvascular occlusion syndrome [25].It is crucial to limit the severity of MI, delay ventricular remodeling, and reduce the occurrence of HF after MI [26]. After MI, the burden on viable cardiomyocytes increases, leading to structural changes in the ventricle, formation of myocardial collagen scarring and myocardial fibrosis, and ultimately myocardial remodeling. Various mechanisms contribute to the development of myocardial remodeling [27]. Although ACEIs have become the standard treatment for chronic HF and the reversal of cardiac remodeling after AMI, Rathod et al. [28] found that enalapril did not improve patient survival rates after MI, whereas ARNI significantly improved the survival rate of patients with AMI. Increased soluble neprilysin in patients with LVEF < 40% may be associated with a higher risk of all-cause death suggesting the potential beneficial effects of ARNI on AMI patients [29]. At present, PCI, CABG, and thrombolytic therapy are the primary methods of treating myocardial ischemia–reperfusion injury. Based on the statistical results of this study, the LVEF of patients treated with ARNI increased to a certain extent, indicating that ARNI improved cardiac function and delayed myocardial remodeling, but did not differ significantly from that of the patients treated with ACEIs.

The present study has two main limitations. First, the sample size of this trial was relatively small. Therefore, future studies on the effect of ARNI treatment in patients with STEMI should incorporate larger sample sizes. Second, this study used relatively few variables to evaluate ventricular remodeling, which could have influenced our results.

In conclusion, in patients with STEMI and reduced LVEF, no significant difference was noted in the short-term improvement of cardiac function with ARNI vs. ACEI therapies. These results are of great significance to the future treatment of STEMI, serving as a reference point for the clinical application of ARNI.

## Supplementary Information

**Additional file 1: Supplement Figure 1.** The flowchart of the patient enrollment. **Supplemental Table 1.** The Multivariate logistic regression analysist of Primary cohort. **Supplemental Table 2.** The Multivariate logistic regressionanalysist of PSM cohort.

## Data Availability

The datasets used and/or analysed during the current study are available from the corresponding author upon reasonable request.

## References

[1] Qureshi WT, Zhang ZM, Chang PP, Rosamond WD, Kitzman DW, Wagenknecht LE, Soliman EZ (2018). Silent Myocardial Infarction and Long-Term Risk of Heart Failure: The ARIC Study. J Am Coll Cardiol.

[2] Rodrigues G, Tralhão A, Aguiar C, Freitas P, Ventosa A, Mendes M (2018). Is the PARADIGM-HF cohort representative of the real-world heart failure patient population?. Rev Port Cardiol.

[3] Weir RA, McMurray JJ, Velazquez EJ (2006). Epidemiology of heart failure and left ventricular systolic dysfunction after acute myocardial infarction: prevalence, clinical characteristics, and prognostic importance. Am J Cardiol.

[4] Gheorghiade M, Zannad F, Sopko G, Klein L, Piña IL, Konstam MA, Massie BM, Roland E, Targum S, Collins SP (2005). Acute heart failure syndromes: current state and framework for future research. Circulation.

[5] Fonarow GC, Adams KF, Abraham WT, Yancy CW, Boscardin WJ (2005). Risk stratification for in-hospital mortality in acutely decompensated heart failure: classification and regression tree analysis. JAMA.

[6] Kompa AR, Lu J, Weller TJ, Kelly DJ, Krum H, von Lueder TG, Wang BH (2018). Angiotensin receptor neprilysin inhibition provides superior cardioprotection compared to angiotensin converting enzyme inhibition after experimental myocardial infarction. Int J Cardiol.

[7] Desai AS, Solomon SD, Shah AM, Claggett BL, Fang JC, Izzo J, McCague K, Abbas CA, Rocha R, Mitchell GF (2019). Effect of sacubitril-valsartan vs enalapril on aortic stiffness in patients with heart failure and reduced ejection fraction: a randomized clinical trial. JAMA.

[8] Zornoff LA, Paiva SA, Duarte DR, Spadaro J (2009). Ventricular remodeling after myocardial infarction: concepts and clinical implications. Arq Bras Cardiol.

[9] Murphy SP, Prescott MF, Camacho A, Iyer SR, Maisel AS, Felker GM, Butler J, Piña IL, Ibrahim NE, Abbas C (2021). Atrial Natriuretic Peptide and Treatment With Sacubitril/Valsartan in Heart Failure With Reduced Ejection Fraction. JACC Heart failure.

[10] Morley JE (2016). Frailty and Sarcopenia: The New Geriatric Giants. Rev Invest Clin.

[11] Lee SE, Lee HY, Cho HJ, Choe WS, Kim H, Choi JO, Jeon ES, Kim MS, Kim JJ, Hwang KK (2017). Clinical characteristics and outcome of acute heart failure in korea: results from the Korean Acute Heart Failure Registry (KorAHF). Korean Circ J.

[12] James PA, Oparil S, Carter BL, Cushman WC, Dennison-Himmelfarb C, Handler J, Lackland DT, LeFevre ML, MacKenzie TD, Ogedegbe O (2014). 2014 evidence-based guideline for the management of high blood pressure in adults: report from the panel members appointed to the Eighth Joint National Committee (JNC 8). JAMA.

[13] Keeley EC, Boura JA, Grines CL (2003). Primary angioplasty versus intravenous thrombolytic therapy for acute myocardial infarction: a quantitative review of 23 randomised trials. Lancet (London, England).

[14] Steg PG, Juliard JM (2005). Primary percutaneous coronary intervention in acute myocardial infarction: time, time, and time!. Heart.

[15] Fahrni G, Wolfrum M, De Maria GL, Cuculi F, Dawkins S, Alkhalil M, Patel N, Forfar JC, Prendergast BD, Choudhury RP (2017). Index of Microcirculatory Resistance at the Time of Primary Percutaneous Coronary Intervention Predicts Early Cardiac Complications: Insights From the OxAMI (Oxford Study in Acute Myocardial Infarction) Cohort. J Am Heart Assoc.

[16] Steg PG, James SK, Atar D, Badano LP, Blömstrom-Lundqvist C, Borger MA, Di Mario C, Dickstein K, Ducrocq G, Fernandez-Aviles F (2012). ESC Guidelines for the management of acute myocardial infarction in patients presenting with ST-segment elevation. Eur Heart J.

[17] Shahin M, Obeid S, Hamed L, Templin C, Gamperli O, Nietlispach F, Maier W, Yousif N, Mach F, Roffi M (2017). Occurrence and Impact of Time Delay to Primary Percutaneous Coronary Intervention in Patients With ST-Segment Elevation Myocardial Infarction. Cardiol Res.

[18] Tschope C, Alogna A, Spillmann F, Faragli A, Schmidt G, Blaschke F, Kuhl U, Hertel E, Willner M, Morris D (2018). The CardioMEMS system in the clinical management of end-stage heart failure patients: three case reports. BMC Cardiovasc Disord.

[19] Mone P, Pansini A, Varzideh F, de Donato A, Jankauskas SS, Santulli G (2022). Exosome-Mediated Angiogenesis Underlies LVAD-Induced Bleeding in Patients With End-Stage Heart Failure. JACC Basic Transl Sci.

[20] Yildiz I, Rencuzogullari I, Karabag Y, Karakayali M, Artac I, Gurevin MS (2022). Predictors of left ventricular ejection function decline in young patients with ST-segment elevation myocardial infarction. Rev Assoc Med Bras (1992).

[21] St John Sutton M, Pfeffer MA, Plappert T, Rouleau JL, Moyé LA, Dagenais GR, Lamas GA, Klein M, Sussex B, Goldman S (1994). Quantitative two-dimensional echocardiographic measurements are major predictors of adverse cardiovascular events after acute myocardial infarction. The protective effects of captopril. Circulation.

[22] Bolognese L, Neskovic AN, Parodi G, Cerisano G, Buonamici P, Santoro GM, Antoniucci D (2002). Left ventricular remodeling after primary coronary angioplasty: patterns of left ventricular dilation and long-term prognostic implications. Circulation.

[23] Farag EM, Al-Daydamony MM (2017). Symptom-to-balloon time and myocardial blush grade are predictors of left ventricular remodelling after successful primary percutaneous coronary intervention. Cardiovasc J Afr.

[24] Liu H, Fu L, Sun X, Peng W, Chen Z, Li Y (2018). Remote ischemic conditioning improves myocardial parameters and clinical outcomes during primary percutaneous coronary intervention: a meta-analysis of randomized controlled trials. Oncotarget.

[25] Rathod KS, Hamshere S, Khambata RS, Andiapen M, Westwood M, Mathur A, Ahluwalia A, Jones DA (2017). Combined analysis of the safety of intra-coronary drug delivery during primary percutaneous coronary intervention for acute myocardial infarction: A study of three clinical trials. JRSM Cardiovasc Dis.

[26] Ong SB, Hernández-Reséndiz S, Crespo-Avilan GE, Mukhametshina RT, Kwek XY, Cabrera-Fuentes HA, Hausenloy DJ (2018). Inflammation following acute myocardial infarction: Multiple players, dynamic roles, and novel therapeutic opportunities. Pharmacol Ther.

[27] Zhai Y, Ao L, Cleveland JC, Zeng Q, Reece TB, Fullerton DA, Meng X (2015). Toll-like receptor 4 mediates the inflammatory responses and matrix protein remodeling in remote non-ischemic myocardium in a mouse model of myocardial ischemia and reperfusion. PLoS ONE.

[28] Ishii M, Kaikita K, Sato K, Sueta D, Fujisue K, Arima Y, Oimatsu Y, Mitsuse T, Onoue Y, Araki S (2017). Cardioprotective Effects of LCZ696 (Sacubitril/Valsartan) After Experimental Acute Myocardial Infarction. JACC Basic Transl Sci.

[29] Choi IJ, Lim S, Hwang Y, Lee D, Lee WJ, Lee KY, Kim MJ, Jeon DS (2020). Soluble neprilysin and long-term clinical outcomes in patients with coronary artery disease undergoing percutaneous coronary intervention: a retrospective cohort study. BMC Cardiovasc Disord.

