# Supplementary material for: Short-term efficacy of angiotensin receptor-neprilysin inhibitor treatment in patients with ST-segment elevation myocardial infarction with reduced ejection fraction after primary percutaneous coronary intervention: a propensity score matching study
Source: BMC Cardiovasc Disord. 2022 Nov 4;22:463. doi: 10.1186/s12872-022-02906-0 (PMC9636690; doi:10.1186/s12872-022-02906-0)
Supplement: Supplementary file 1 — Additional file 1: Supplement Figure 1. The flowchart of the patient enrollment. Supplemental Table 1. The Multivariate logistic regression analysist of Primary cohort. Supplemental Table 2. The Multivariate logistic regressionanalysist of PSM cohort. [file 12872_2022_2906_MOESM1_ESM.docx]

Supplemental Data


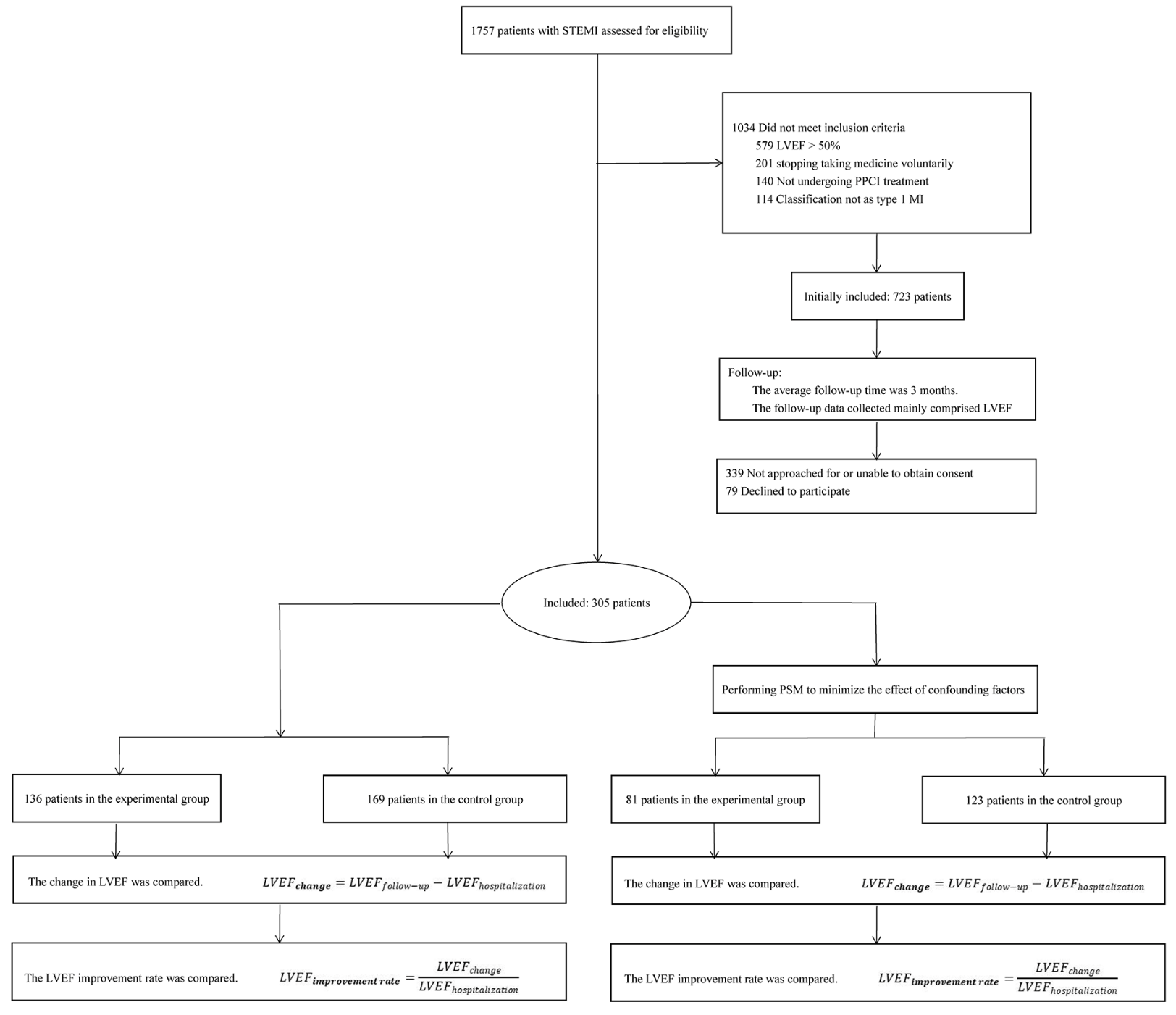


Supplement Figure 1. The flowchart of the patient enrollment.

## Supplemental Table1. The Multivariate logistic regression analysist of Primary cohort.

|  | Variable | B | SE | Wald | *P* | OR | 95％*CI* |
| --- | --- | --- | --- | --- | --- | --- | --- |
| Lower | Intercept | -1.329 | 2.944 | 0.204 | 0.652 |  |  |
|  | Age | 0.071 | 0.045 | 2.478 | 0.115 | 1.073 | 0.983-1.172 |
|  | NT-proBNP | 0 | 0 | 0.614 | 0.433 | 1 | 1 |
|  | CTNI | 0 | 0 | 0.604 | 0.437 | 1 | 1 |
|  | ARNI |  |  |  |  |  |  |
|  | NO | -0.951 | 0.737 | 1.665 | 0.197 | 0.386 | 0.091-1.638 |
|  | YES | Ref. |  |  |  |  |  |
|  | Sex |  |  |  |  |  |  |
|  | Male | 0.234 | 0.67 | 0.122 | 0.727 | 1.263 | 0.34-4.693 |
|  | Female | Ref. |  |  |  |  |  |
|  | Hypertension |  |  |  |  |  |  |
|  | No | -1.079 | 0.626 | 2.97 | 0.085 | 0.34 | 0.1-1.16 |
|  | Yes | Ref. |  |  |  |  |  |
|  | Diabetes |  |  |  |  |  |  |
|  | No | -0.126 | 0.672 | 0.035 | 0.852 | 0.882 | 0.236-3.294 |
|  | Yes | Ref. |  |  |  |  |  |
|  | Smoking history |  |  |  |  |  |  |
|  | No smoking | -1.252 | 0.769 | 2.651 | 0.103 | 0.286 | 0.063-1.291 |
|  | Quit smoking | 16.177 | 2830.548 | 0 | 0.995 | 10601812.24 | 0 |
|  | Still smoking | Ref. |  |  |  |  |  |
|  | Drinking history |  |  |  |  |  |  |
|  | No drinking | 0.568 | 0.624 | 0.829 | 0.363 | 1.766 | 0.519-6.002 |
|  | Quit drinking | 17.467 | 4383.85 | 0 | 0.997 | 38523224.81 | 0 |
|  | Still drinking | Ref. |  |  |  |  |  |
|  | β-Receptor blocker |  |  |  |  |  |  |
|  | No | 0.104 | 1.239 | 0.007 | 0.933 | 1.11 | 0.098-12.586 |
|  | Yes | Ref. |  |  |  |  |  |
|  | Spironolactone |  |  |  |  |  |  |
|  | No | -0.039 | 0.749 | 0.003 | 0.958 | 0.962 | 0.222-4.171 |
|  | Yes | Ref. |  |  |  |  |  |
|  | Statins |  |  |  |  |  |  |
|  | No | 17.847 | 0.723 | 608.915 | 0 | 56345041.07 | 13652946.83-232533217.3 |
|  | Yes | Ref. |  |  |  |  |  |
| Higher | Intercept | 4.196 | 2.804 | 2.239 | 0.135 |  |  |
|  | Age | -0.008 | 0.043 | 0.038 | 0.845 | 0.992 | 0.912-1.079 |
|  | NT-proBNP | 0 | 0 | 1.228 | 0.268 | 1 | 1 |
|  | CTNI | 0 | 0 | 0.559 | 0.455 | 1 | 1 |
|  | ARNI |  |  |  |  |  |  |
|  | NO | -0.166 | 0.715 | 0.054 | 0.816 | 0.847 | 0.208-3.441 |
|  | YES | Ref. |  |  |  |  |  |
|  | Sex |  |  |  |  |  |  |
|  | Male | 0.095 | 0.644 | 0.022 | 0.883 | 1.099 | 0.311-3.883 |
|  | Female | Ref. |  |  |  |  |  |
|  | Hypertension |  |  |  |  |  |  |
|  | No | -0.983 | 0.605 | 2.636 | 0.104 | 0.374 | 0.114-1.226 |
|  | Yes | Ref. |  |  |  |  |  |
|  | Diabetes |  |  |  |  |  |  |
|  | No | -0.053 | 0.649 | 0.007 | 0.935 | 0.948 | 0.266-3.382 |
|  | Yes | Ref. |  |  |  |  |  |
|  | Smoking history |  |  |  |  |  |  |
|  | No smoking | -1.219 | 0.743 | 2.69 | 0.101 | 0.295 | 0.069-1.268 |
|  | Quit smoking | 16.201 | 2830.548 | 0 | 0.995 | 10862779.95 | 0 |
|  | Still smoking | Ref. |  |  |  |  |  |
|  | Drinking history |  |  |  |  |  |  |
|  | No drinking | 0.248 | 0.601 | 0.171 | 0.679 | 1.282 | 0.395-4.159 |
|  | Quit drinking | 17.412 | 4383.85 | 0 | 0.997 | 36456181.62 | 0 |
|  | Still drinking | Ref. |  |  |  |  |  |
|  | β-Receptor blocker |  |  |  |  |  |  |
|  | No | -0.305 | 1.214 | 0.063 | 0.802 | 0.737 | 0.068-7.962 |
|  | Yes | Ref. |  |  |  |  |  |
|  | Spironolactone |  |  |  |  |  |  |
|  | No | 0.544 | 0.71 | 0.587 | 0.444 | 1.723 | 0.428-6.929 |
|  | Yes | Ref. |  |  |  |  |  |
|  | Statins |  |  |  |  |  |  |
|  | No | 17.54 | 0 | . | . | 41459784.15 | 41459784.15-41459784.15 |
|  | Yes | Ref. |  |  |  |  |  |

## Supplemental Table 2. The Multivariate logistic regression analysist of PSM cohort.

|  | Variable | B | SE | Wald | *P* | OR | 95％*CI* |
| --- | --- | --- | --- | --- | --- | --- | --- |
| Lower | Intercept | -0.643 | 4.885 | 0.017 | 0.895 |  |  |
|  | Age | 0.051 | 0.069 | 0.549 | 0.459 | 1.053 | 0.919-1.206 |
|  | NT-proBNP | 0 | 0 | 0.986 | 0.321 | 1 | 1 |
|  | CTNI | 0 | 0 | 0.24 | 0.624 | 1 | 1 |
|  | ARNI |  |  |  |  |  |  |
|  | NO | -0.915 | 0.779 | 1.378 | 0.241 | 0.401 | 0.087-1.846 |
|  | YES | Ref. |  |  |  |  |  |
|  | Sex |  |  |  |  |  |  |
|  | Male | 0.091 | 0.822 | 0.012 | 0.912 | 1.095 | 0.219-5.485 |
|  | Female | Ref. |  |  |  |  |  |
|  | Hypertension |  |  |  |  |  |  |
|  | No | -1.121 | 0.739 | 2.302 | 0.129 | 0.326 | 0.077-1.387 |
|  | Yes | Ref. |  |  |  |  |  |
|  | Diabetes |  |  |  |  |  |  |
|  | No | 0.288 | 0.773 | 0.139 | 0.709 | 1.334 | 0.293-6.066 |
|  | Yes | Ref. |  |  |  |  |  |
|  | Smoking history |  |  |  |  |  |  |
|  | No smoking | -0.829 | 0.835 | 0.986 | 0.321 | 0.437 | 0.085-2.242 |
|  | Quit smoking | 16.352 | 3063.589 | 0 | 0.996 | 12630209.94 | 0 |
|  | Still smoking | Ref. |  |  |  |  |  |
|  | Drinking history |  |  |  |  |  |  |
|  | No drinking | 1.19 | 0.748 | 2.532 | 0.112 | 3.286 | 0.759-14.221 |
|  | Quit drinking | 17.292 | 4854.201 | 0 | 0.997 | 32342182.22 | 0 |
|  | Still drinking | Ref. |  |  |  |  |  |
|  | β-Receptor blocker |  |  |  |  |  |  |
|  | No | 0.779 | 1.493 | 0.272 | 0.602 | 2.18 | 0.117-40.694 |
|  | Yes | Ref. |  |  |  |  |  |
|  | Spironolactone |  |  |  |  |  |  |
|  | No | -0.51 | 0.843 | 0.367 | 0.545 | 0.6 | 0.115-3.132 |
|  | Yes | Ref. |  |  |  |  |  |
|  | Statins |  |  |  |  |  |  |
|  | No | 18.152 | 0.921 | 388.466 | 0 | 76474452.11 | 12576515.48-465020842.6 |
|  | Yes | Ref. |  |  |  |  |  |
| Higher | Intercept | 4.954 | 4.656 | 1.132 | 0.287 |  |  |
|  | Age | -0.028 | 0.066 | 0.179 | 0.673 | 0.973 | 0.855-1.107 |
|  | NT-proBNP | 0 | 0 | 1.558 | 0.212 | 1 | 1 |
|  | CTNI | 0 | 0 | 0.382 | 0.536 | 1 | 1 |
|  | ARNI |  |  |  |  |  |  |
|  | NO | -0.186 | 0.765 | 0.059 | 0.808 | 0.831 | 0.185-3.719 |
|  | YES | Ref. |  |  |  |  |  |
|  | Sex |  |  |  |  |  |  |
|  | Male | -0.298 | 0.798 | 0.139 | 0.709 | 0.743 | 0.155-3.551 |
|  | Female | Ref. |  |  |  |  |  |
|  | Hypertension |  |  |  |  |  |  |
|  | No | -0.94 | 0.718 | 1.713 | 0.191 | 0.391 | 0.096-1.596 |
|  | Yes | Ref. |  |  |  |  |  |
|  | Diabetes |  |  |  |  |  |  |
|  | No | 0.577 | 0.755 | 0.584 | 0.445 | 1.781 | 0.405-7.824 |
|  | Yes | Ref. |  |  |  |  |  |
|  | Smoking history |  |  |  |  |  |  |
|  | No smoking | -1.186 | 0.809 | 2.151 | 0.142 | 0.305 | 0.063-1.49 |
|  | Quit smoking | 16.299 | 3063.589 | 0 | 0.996 | 11987887.09 | 0 |
|  | Still smoking | Ref. |  |  |  |  |  |
|  | Drinking history |  |  |  |  |  |  |
|  | No drinking | 0.87 | 0.725 | 1.442 | 0.23 | 2.388 | 0.577-9.88 |
|  | Quit drinking | 17.617 | 4854.201 | 0 | 0.997 | 44775753.19 | 0 |
|  | Still drinking | Ref. |  |  |  |  |  |
|  | β-Receptor blocker |  |  |  |  |  |  |
|  | No | 0.076 | 1.479 | 0.003 | 0.959 | 1.079 | 0.059-19.564 |
|  | Yes | Ref. |  |  |  |  |  |
|  | Spironolactone |  |  |  |  |  |  |
|  | No | 0.059 | 0.804 | 0.005 | 0.942 | 1.06 | 0.219-5.125 |
|  | Yes | Ref. |  |  |  |  |  |
|  | Statins |  |  |  |  |  |  |
|  | No | 17.506 | 0 | . | . | 40057605.32 | 40057605.32-40057605.32 |
|  | Yes | Ref. |  |  |  |  |  |
